# Effect of strigolactone on growth, photosynthetic efficiency, antioxidant activity, and osmolytes accumulation in different maize (*Zea mays* L.) hybrids grown under drought stress

**DOI:** 10.1080/15592324.2023.2262795

**Published:** 2023-12-17

**Authors:** Muhammad Luqman, Muhammad Shahbaz, Muhammad Faisal Maqsood, Fozia Farhat, Usman Zulfiqar, Manzer H. Siddiqui, Atifa Masood, Muhammad Aqeel, Fasih Ullah Haider

**Affiliations:** a Department of Botany, University of Agriculture, Faisalabad, Pakistan; b Department of Botany, The Islamia University of Bahawalpur, Bahawalpur, Pakistan; c Department of Botany, Government College Women University, Faisalabad, Pakistan; d Department of Agronomy, Faculty of Agriculture and Environment, The Islamia University of Bahawalpur, Bahawalpur, Pakistan; e Department of Botany and Microbiology, College of Science, King Saud University, Riyadh, Saudi Arabia; f The department of Botany, University of Lahore, Sargodha, Pakistan; g State key Laboratory of Grassland Agro-Ecosystems, School of Life Sciences, Lanzhou University, Lanzhou, China; h Ecology, Key Laboratory of Vegetation Restoration and Management of Degraded Ecosystems, South China Botanical Garden, Chinese Academy of Sciences, Guangzhou, China; i Ecology, University of Chinese Academy of Sciences, Beijing, China

**Keywords:** Strigolactones, drought, maize, proline, glycinebetaine

## Abstract

Drought alters plant physiology, morphology, and biochemical pathways, necessitating effective mitigation strategies. Strigolactones (SLs) are phytohormones known to enhance plant growth under abiotic stress. However, their specific impact on drought stress in maize remains unclear. This study aimed to determine the optimal SL concentration for mitigating drought stress in two maize hybrids (HY-1898, FH-1046). Maize plants were subjected to 60% field capacity drought stress in a pot experiment. After 40 d, different concentrations (0, 0.001, 0.01, and 0.1 mg L^−1^) of the synthetic SL analogue GR24 were applied to evaluate their effects on growth features, photosynthesis attributes, and osmolyte accumulation in the maize hybrids. Results showed that exogenous SL application significantly increased photosynthetic pigments in maize hybrids under drought stress. Chlorophyll content, gas exchange characteristics, net CO_2_ assimilation rate, stomatal conductance, water use efficiency, and antioxidant activities were enhanced by GR24. Leaf ascorbic acid and total phenolics also increased with SL application. Organic osmolytes, such as glycine betaine and free proline, were elevated in both maize hybrids under drought stress. Yield-related parameters, including cob diameter, cob weight, number of seeds per cob, and number of seeds per plant, were significantly increased by GR24 under drought stress. Our findings highlight the potential of GR24 foliar application to mitigate drought stress and promote maize growth and grain yield in a concentration-dependent manner. The minimum effective SL concentration against drought stress was determined to be 0.01 mg L^−1^. Overall, foliar application of GR24 could serve as a sustainable approach for drought tolerance in agriculture.

## 1. Introduction

Global agricultural production and consumption in 2050 are projected to be 60% higher than in 2005/07sa.^1^ It is also conjectured that by the year 2050 world population will be projected to be up to 9.7 billion, with an average of ~65% of individuals depending directly or indirectly on agriculture for their livelihood. This percentage will likely rise to 90% in poor, less developed countries.^2^ Consequently, not only a matter of nation’s economy but surely the food supply is perhaps largely dependent on agriculture. However, agricultural practices encounter many problems, such as inaccessibility of an adequate irrigation system, small and fragmented landholdings, unavailability of good-quality seeds, lack of proper mechanization and over usage of chemical fertilizers and pesticides. All these lead to unfit soils, soil erosion and natural calamities.^3^

The perceived limit to producing food for a growing global population remains a source of debate and preoccupation despite the agriculture sector’s historical ability to meet such demand.^4^ Abiotic stresses like heavy metals, salinity, water scarcity, high ambient temperature, etc., are the most significant limiting factors of plant survival and growth under the increasing crisis of climatic changes.^5^ These environmental factors are alarming the world’s food security and deterioration of the nutritional quality of significant agricultural products. The abrupt change in natural factors due to anthropogenic activities further decreases the water sources, consequently causing drought stress in major agro-systems of the world, especially in rain-fed ecosystems.^6^ It has been estimated that freshwater availability has decreased by 20% and that 3.2 billion people stay in high to extremely high water deficit conditions.^7^ Besides this, available water quality is also deteriorating in these areas. Agriculture is most vulnerable to water scarcity among many enlisted sectors and faces a significant decline in yield potential (40–60%) in rain-fed areas.^8^

Drought is one of the mainstream environmental constrain limiting plant vigor and development.^9^ With the revealing global climate change, the frequency and intensity of regional droughts are also increasing yearly.^10^ On the one hand, drought can reduce the photosynthesis rate of leaves and destroy plant photosynthesis organs, leading to plants’ inability to effectively utilize absorbed light energy.^11,12^ On the other hand, drought can affect nutrient acquisition, transport, distribution, and storage, leading to decreased plant-biomass accumulation and root vigor.^13,14^ Plant hormones play a crucial role in regulating plant responses to abiotic stresses.^15^ Drought stress damages many plants’ morphological, physiological, and biochemical processes by disturbing the stomatal closure, ultrastructure of leaves, and cellular dehydration. Moreover, it triggers members’ lipid peroxidation, reducing the antioxidant ability and endogenous hormones.^13,14^ Farooq et al.^15^ reported that drought causes serious effects on crop yield reduction, such as *Hordeum vulgare* (barley; 49–57%), *Zea mays* (maize; 47–70%), *Cicer arietinum* (chickpea; 24–84%), *Glycine max* (soybean; 46–71%) and *Oryza sativa* (rice; 30–55%).

Maize is an important oil crop in many nations around the globe and is consumed as a staple food in a few parts of the world and industry. Moreover, it can also be used as a raw material for their biofuel.^16^ Therefore, much enormous focus has been given to the maize crop to improve crop resistance against the abiotic and biotic stresses.^17^ Drought stress damages the cellular organelles, involved in the deformation of protein structure, membrane lipids and cellular compartments through the production of reactive oxygen species (ROS) in bulk concentration.^18^ Water deficit conditions disturb the gaseous exchange characteristics of plants and have deteriorated impacts on the physiology and yield of maize plants.^19^ Water stress in maize decreased yield by delaying silking and lengthening the time from anthesis to silking. This characteristic was closely associated with grain yield, depending on the number of ears and kernels per plant.^15^

Therefore, water utilization is a worthy task to manage food security and ensure zero hunger around the globe. The researchers are consistently working on this issue and have achieved many milestones like developing a water storage facility for rainwater harvesting, maintaining an efficient water ecosystem, water-saving agriculture, improving water use efficiency and use of water conservation techniques.^20,21^ However, the existing approaches and technology are insufficient to achieve the future goal of zero hunger; therefore, drought stress has emerged as a major abiotic stress in the agriculture system. Strigolactones (SLs) are carotenoid-derived molecules that influence plants’ growth and developmental processes and adaptation mechanisms. These are significantly involved in plants’ root and shoot architecture, leaf senescence, etc. Plants grown under nutrient-deprived conditions, especially phosphorus-deficient soil, induced SL production and facilitated the root architecture and arbuscular mycorrhizal association in the rhizosphere, which is responsible for increasing plant nutrient uptake.^22^ In drought and salt stress regimes, transcriptomic analysis in *Arabidopsis* explored that SLs positively regulate the hypersensitivity responses.^23^ In rice mutants, docking of SLs production decreases the plant length and biomass but increases tillers production.^24^ GR24 has been used extensively among various synthetic analogues in plants because of its potential role in mitigating abiotic stresses.^25^ GR24 is a synthetic analogue of SLs used to investigate the role of SL under drought regime.^26^ Considering the significance of *Z. mays* L. and the distressing impacts of water scarcity on crop production and yield, contemporary study directs to develop an environment-friendly technique. Maize (*Zea mays* L.) is a staple food of many world countries and plays a substantial role in coping with regional food security. The average maize yield in tropical Asia is low compared to developed temperate regions of America.^6^ Maize is susceptible to shortage of water, and a 39.3% decline in grain yield occurred in maize due to a 40% limited water supply.^27^ So, under such a scenario, water deficit regimes are the most hazardous factors that limit growth in maize.^28^ The underlying mechanism of strigolactones (GR24) in reducing drought stress in crops, notably in hybrid maize, has rarely been studied, in contrast to the advantages of SLs under other abiotic stress conditions. To our knowledge, very few research have comprehensively examined how SLs help plants stressed by drought. Whether foliar SLs (GR24) application increases stress tolerance is little known. In the current study, we sought to assess how SLs (GR24) affected hybrid maize plant development and the gas exchange attributes and maize yield-related parameters in reducing drought stress. Interplay between SLs (GR24) and maize stress tolerance mechanisms, such as the osmolytes production, were also examined in response to drought. It was assumed that the foliar application of SLs (GR24) helps to mitigate drought stress in various maize hybrids by improving chlorophyll contents, gas exchange activity, and osmolyte production in plants grown under drought stress.

## 2. Materials and methods

### 2.1. GR24 and seeds procurement

GR24 (M. wt. 298.2), a synthetic analogue of strigolactones, was purchased from the Organic Chemistry Department, Radboud University Nijmegen, Netherlands, and seeds of maize hybrids, FH-1046 and HY-1898, were attained from Maize Research Center Yousafwala, district Sahiwal, Punjab, Pakistan. The maize hybrid YH-1898 was released in 2016 via single cross, with an average yield of 10,300 Kg/ha. Similarly, FH-1046 was released in 2017 via single cross, with an average yield of 11,000 Kg/ha (https://aari.punjab.gov.pk/).

### 2.2. Experimental design and setup

A pot experiment was conducted in the net house of Old Botanical Garden, Department of Botany, University of Agriculture Faisalabad (31° 25‘N, 73° 05’ E), Pakistan. The soil used in this study was collected from the Agronomic Research farm, Department of Agronomy, UAF. The experimental soil had an electrical conductivity of 1.35 dS m^−1^, pH 7.7, organic matter content of 0.98%, available phosphorus of 7.8 mg kg^−1^, available potassium of 160 mg kg^−1^, and water saturation of 42% with sandy-loam soil texture. The current study was a three-factor factorial experiment having eight treatments with three replicates (total treatments 24) under a completely randomized design (CRD). Each plastic pot was (diameter 25 cm and height 28 cm) filled with 6 kg soil, and eight seeds of both maize hybrids were sown in each plastic pot. The sterilized maize seeds were allowed to germinate for 28 d and maintained five plants pot pot by thinning. Experimental pots were dissected into two groups, i.e., drought-stressed plants having 60% field capacity (FC) and non-stressed plants (normal watering). The 60% FC was calculated by weighing the fresh and dry weight of the soil in the pot, and later moisture contents were measured in 100 g oven-dry soil samples by applying the standard formula. Moisture concentration levels of drought-stressed pots were examined frequently by maintaining the moisture level in each pot according to the calculated value of 60% FC through proper watering. Thereafter, calculate the FC % by the given formula as equation 1.

(1) $$FC\%=\;\left(Iw-Fw/Fw\right)*100$$

After 20 d of drought application, both groups of plants were further dissected into four categories for GR24 foliar application. The GR24 stock solution of 0.2 mg L^−1^ was prepared in 1000 mL of distilled water. The desired concentration (0, 0.001, 0.01, and 0.1 mg L^−1^) was diluted from the stock solution, each dilution was mixed with 1% tween-20 as surfactant. All the treatments were performed once a week till the record of data. The data of different parameters were collected after three weeks of foliar application. Weeding of whole experimental setup was done by hands. The average maximum and minimum temperatures during the experiment were 29 and 15°C, respectively, relative humidity was from 59 to 73%, and the average rainfall was 1.15 mm.

### 2.3. Chlorophyll estimation

Chlorophyll (*Chl*) pigments and carotenoid contents of maize leaves were examined following the Arnon,^29^ procedure with minor modifications. Fresh leaves (0.1 g) of each replicate were chopped down into small fragments, added into 5 ml of 80% acetone in 10 ml plastic bottles and incubated at 4°C in the dark overnight. The supernatant was used to record absorbance with the help of a spectrophotometer (IRMECO U2020, Geesthacht/Germany) at 663, 645, and 480 nm.

### 2.4. Gas exchange characteristics

Gas exchange parameters were investigated using LCA-4 ADC transportable infrared gas analyzer [IRGA (LCA-4) Analytical Development Company, Hoddesdon, England] from maize leaves from 10 am to 11 am. Environmental conditions given by IRGA during data collection were; the gas flow of the leaf chamber was 260 µmol, ambient concentration of carbon dioxide during data collection was 402.1 µmol mol^−1^, the temperature of the leaf chamber fluctuated between 25–30°C and PAR was examined maximum up to 900 µmol m^−2^ s^−1^. The following parameters were examined; net carbon dioxide assimilation rates (*A*), stomatal conductance (g_s_), transpiration rate (*E*), internal carbon dioxide concentration (*C*_*i*_), water use efficiency (*A/E*) and *C*_*i*_*/C*_*a*_ were also examined according to the procedure mentioned by Maqsood et al.^30^

### 2.5. Antioxidant activities

Enzymatic antioxidant extraction involved homogenizing 0.5 g of fresh leaf material in a potassium phosphate buffer (K_2_HPO_4_, KH_2_PO_4_) solution (10 ml). The homogenate was then centrifugated at 15,000 × *g* for 15 min, and the resulting supernatant was utilized to estimate antioxidant activities. Catalase (CAT) and peroxidase (POD) activity were determined according to the protocol mentioned by Maqsood et al.^31^ The sample mixture consisted of enzyme extract (0.1 µL), potassium buffer (1.9 mL), and H_2_O_2_ (1 mL). Absorbance values were measured using a spectrophotometer (IRMECO U-2020 Lütjensee, Germany) at 240 nm for a duration of 120 s with an interval of every 30 s. Similarly, the POD reaction mixture was prepared by combining guaiacol (100 µL), potassium buffer (750 µL), enzyme extract (100 µL), and H_2_O_2_ (100 µL). Readings were then recorded at 470 nm using a spectrophotometer (IRMECO U-2020 Lütjensee, Germany) for 2 min with an interval of 30 s. The approach described by Giannopolitis and Ries^32^ procedure was followed to estimate superoxide dismutase (SOD) activity. The reaction mixture consisted of distilled/deionized water (400 µL), methionine (100 µL), enzyme extract (50 µL), triton (100 µL), phosphate buffer (250 µL), nitroblue tetrazolium (50 µL) and riboflavin (50 µL). Subsequently, the mixture was exposed to light (60 watts) for 20 min, and readings were taken using a spectrophotometer (IRMECO U-2020 Lütjensee, Germany) at 560 nm.

### 2.6. Oxidant activities

The fresh leaf material weighing 0.5 g was macerated in 0.1% TCA (5 mL) following the method described by Maqsood et al.^33^ After centrifugation, a 0.5 mL sample mixture was prepared by adding potassium iodide solution (1 mL) and potassium phosphate buffer (500 µL). The mixture was vortexed for 5–7 s, and values were determined using a spectrophotometer (IRMECO U-2020 Lütjensee, Germany) at 390 nm. The malondialdehyde (MDA) contents were measured following the protocol outlined by Cakmak and Horst (1991). Fresh maize leaves (0.5 g) were ground in a solution of TCA (10 mL) and centrifuged at 12,000 × *g* for 15 min. To 1 mL of the resulting sample mixture, 4 mL of 0.5% TBA (4 mL) was added and placed in a water bath at 95°C for 30 min. Readings were taken at 532 and 600 nm using a spectrophotometer (IRMECO U-2020 Lütjensee, Germany) against a blank consisting of 5% TCA. The EL was determined by taking small pieces of maize leaves (0.25 g) in the tube containing 20 mL of distilled water. The initial EC was noted, and the tube was incubated at 100°C for 0.5 h. Then, the final EC of the solution in the tube was reported. The EL of maize leaves was recorded as described by Maqsood et al.^24^ For the determination of leaf H_2_O_2_ contents, leaf samples (50 mg) were added into 50 mM phosphate buffer (3 mL) with pH 6.5. The reaction mixture was centrifuged at 6000 × *g* for 30 min at 4°C. After centrifugation, 1 mL of 0.1% Titanium sulfate (O_8_S_2_Ti) having H_2_SO_4_ (20%, v/v) was added to the solution mixture. The reaction mixture was centrifuged at 6000 × *g* for 20 min at 4°C. The absorption of the supernatant was observed at 410 nm wavelength.

### 2.7. Leaf ascorbic acid and total phenolics estimation

Mukherjee and Choudhury,^34^ method was used to determine the ascorbic acid contents in both maize hybrids. Briefly, a fresh leaf sample (250 mg) was ground in 5 mL of 6% trichloroacetic acid (TCA) by using a pestle and mortar. Later, centrifuged at 10,000 rpm for 20 min. Dissolved 0.5 mL of Dinitrophenyl hydrazine (in acidic medium) in supernatant extract (1 mL) followed by the addition of 1 drop of 10% thiourea prepared in 70% ethanol. The mixture was incubated in a water bath for 15 min. and then terminated the reaction was immediately in an ice bath. After cooling, 80% H_2_SO_4_ (1 mL) was added to the mixture. Absorbance was recorded at 530 nm using a spectrophotometer (IRMECO U2020, Geesthacht/Germany).

Ascorbic acid concentration was estimated from a standard curve, plotted with known ascorbic acid concentration, and expressed as mg g-1 fresh weight (FW) of plant biomass. Total soluble phenols were analyzed by Julkunen-Titto^35^ The fresh leaf sample with specific weight was homogenized in 80% acetone and then centrifuged for 15 min at 1000 × g, the supernatant (0.1 mL) was mixed with 2 mL water and 1 mL Folin-Ciocalteau phenol reagent and shaken well. A 10 ml of distilled water was added to 5 ml of 20% Na2CO3. Optical density (OD) at 750 nm was obtained using a spectrophotometer (IRMECO U2020, Geesthacht/Germany).

### 2.8. Glycinebetaine and free proline determination

Glycinebetaine in leaves of both maize hybrid treatments was determined by following the method of Grieve and Grattan.^36^ Dried plant sample of shoot (0.25 g) ground added 5 mL of 0.5% toluene-water mixture. Samples were placed for 24 h at room temperature. Later, it was filtered, 0.5 mL filtrate was treated with 0.5 mL of the 2N-HCl solution, further diluted with 0.1 ml of potassium tri-iodide solution (prepared as 7.5 g iodide and 10 kg KI_2_ in 100 mL of 1N-HCl), shaken in ice cold bath, added 5 mL of chilled 1,2 dichloroethane. It was then vortexed for 20 sec to form two layers. The lower layer was taken for absorbance at 365 nm with a spectrophotometer (IRMECO U2020, Geesthacht/Germany).

Bates et al.^37^ method was used to determine the leaf-free proline contents. Fresh plant leaf sample (500 mg) was homogenized in 5 ml of 3% sulfosalicylic acid in water and homogenate was centrifuged for 20 min. Then, one milliliter (1 mL) of the filtrate was mixed with 1 mL of acidic ninhydrin solution and 1 mL of glacial acetic acid in a test tube. After quenching the reaction in an ice bath, the mixture was heated in a 100°C water bath for 1 h. It was then extracted with toluene (2 mL), the sample was vortexed for 15–20 s, and a continuous stream of air was passed through it for 1 min. Toluene containing the chromophore was aspirated from the aqueous phase, the toluene layer was carefully collected and the absorbance at 520 nm was observed using a spectrophotometer (IRMECO U2020, Geesthacht/Germany) with toluene as a blank.

### 2.9. Yields attributes

At full maturity, the cob weight and cob diameter of each replicate were measured with measuring tape and Vernier caliper, respectively. The number of cobs per plant and number of seeds per cob was determined for each replicate manually.

### 2.10. Statistical analysis

Experimental units were arranged in the three-factor factorial way with three replicates of each treatment according to a completely randomized design (CRD). Data were analyzed by the analysis of variance with Tukey’s test for differences among mean values (*P <* .05) for each parameter and were computed using the COSTAT Computer Program (COSTAT).^29^ The graphs were constructed in Excel (V, 2013), and the principal component analysis (PCA) and correlation were applied through statistical software “R (v 4.0.1)”.

## 3. Results

### 3.1. Exogenous GR24 protected photosynthesis under drought conditions

**Figure 1.**
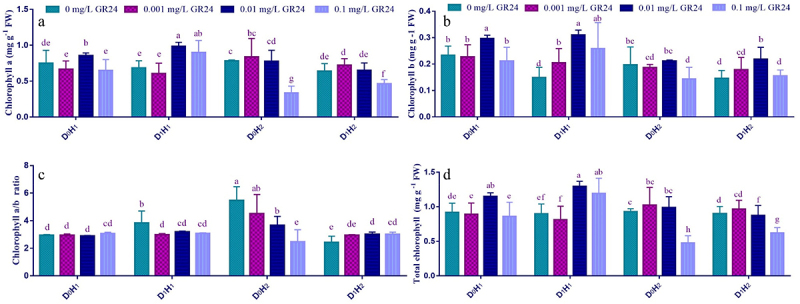
Effect of SL (GR24) application on chlorophyll *a* (a), chlorophyll *b* (b), chlorophyll *a*/*b* (c), and total chlorophyll (d) of two maize hybrids under drought stress. Different lowercase letters indicate significant differences as determined by the LSD test (*p* ≤0.05). Replication = 3; D_0_= no drought control; D_1_= drought stress at 60% field capacity; H_1_= maize hybrid (FH-1046); H_2_= maize hybrid (HY-1898).

Table S1 exhibits the role of GR24 as foliar treatment with varying concentrations on photosynthetic pigments of two maize hybrids (H) that were grown under drought (D) stress. Data of these attributes demonstrates that hybrid maize plant subjecting to drought stress considerably hampered the photosynthetic attributes such as Chl *a*, Chl *b*, and T. Chl contents (Figure 1). The percentage decrease was 11.53%, 36.41%, 11.29% for Chl *a*, Chl *b* and T. Chl contents, respectively, in both hybrids. Regardless of drought behavior, treatment of both maize hybrids with GR24 application enhanced all the above-mentioned photosynthetic parameters at either normal irrigation (D_0_) or drought stress (D_1_), compared with their corresponding controls (Figure 1). Contrary to other photosynthetic attributes, Chl *a*/*b* ratio and carotenoids increased significantly with D_1_ in H_1_ (30.13, 13.11%) and H_2_ (18.60%), respectively. Data clearly display that the most effective concentration of GR24 was 0.01 mg L^−1^, as it caused the greatest increase in photosynthetic attributes, particularly in D_I_H_1_ plant (Figure 1).

**Figure 2.**
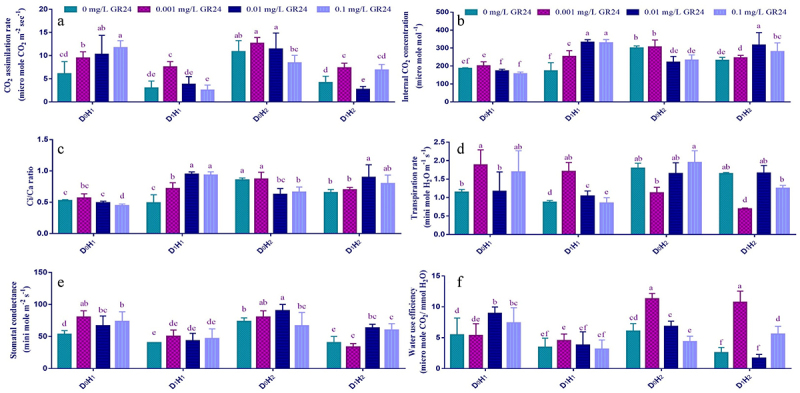
Effect of SL (GR24) application on CO_2_ assimilation rate (a), internal CO_2_ concentration (b), Ci/Ca ratio (c), transpiration rate (d), stomatal conductance (e), and water use efficiency (f) of two maize hybrids under drought stress. Different lowercase letters indicate significant differences as determined by the LSD test (*p* ≤0.05). Replication = 3; D_0_= no drought control; D_1_= drought stress at 60% field capacity; H_1_= maize hybrid (FH-1046); H_2_= maize hybrid (HY-1898).

Gas exchange parameters were recorded to further understand the GR24 impacts on photosynthesis of plants. The foliar application of GR24 under drought regime significantly affects the gas exchange parameters. CO_2_ assimilation rate (*A*), transpiration rate (*E*), water use efficiency (*WUE*) and sub-stomatal conductance (*g*_*s*_), all these attributes were decreased under the water deficit condition, but the foliar application of GR24 reassured these downtrends (Table S1, Figure 2). Irrigation showing 60% FC, caused decreases 50.43, 7.17, 7.73, 23.39, 24.99 and 36.77% in H_1_ maize hybrid, while the percent of decreases was 61.94, 8.88, 23.33, 22.97, 45.45 and 58.12% were in H_2_ maize hybrid for *A* (Figure 2a), *Ci* (Figure 2b), *Ci/Ca* (Figure 2c), *E* (Figure 2d), g_s_ (Figure 2e), and *WUE* (Figure 2f), respectively, as compared with plants with 100% irrigation water requirement (D_0_). On the other hand, foliar treatment of GR24 (0.001, 0.01, 0.1 mg L^−1^) on the two tested maize hybrid plants increased the above-mentioned gas exchange attributes (Figure 2). More precisely, 0.01 and 0.1 mg L^−1^ GR24 considerably enhanced *Ci* (90.13, 30.69%), *Ci/C*_a_ (91.25, 20.50%), g_s_ (12.33, 53.31%) and WUE (2.10%, 2 folds) in H_1_ and H_2_ maize hybrids, respectively, compared to their corresponding control, without GR24 foliar application (0 mg L^−1^) (Figure 2). These results further support the ability of GR24-treated plants to maintain osmolytes accumulation during drought stress.

### 3.2. Changes in MDA, H_2_O_2_, and antioxidants pool under drought stress with GR24 application

**Figure 3.**
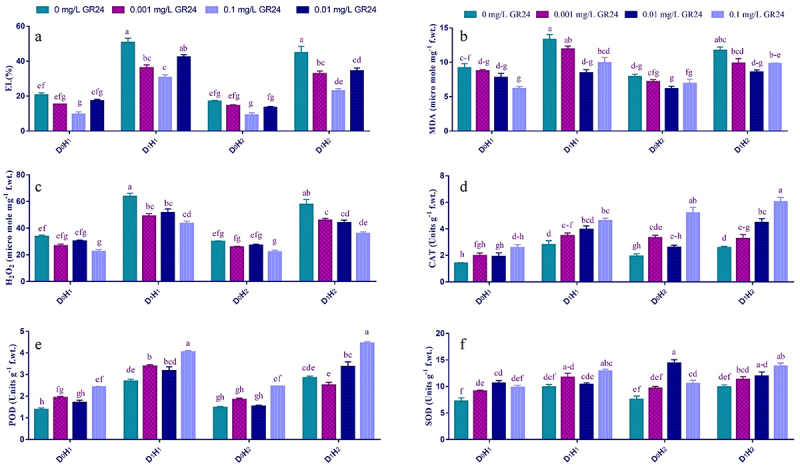
Effect of SL (GR24) application on electrolyte leakage (a), malonaldehyde content (b), hydrogen peroxide (c), catalase (d), peroxidase (e), and superoxide (f) of two maize hybrids leaves under drought stress. Different lowercase letters indicate significant differences as determined by the LSD test (*p* ≤0.05). Replication = 3; D_0_= no drought control; D_1_= drought stress at 60% field capacity; H_1_= maize hybrid (FH-1046); H_2_= maize hybrid (HY-1898).

The end product of lipid peroxidation in the cell membrane is MDA. In our investigation, MDA contents were significantly raised by ~ 58.7%–64.7% in maize seedlings subjected to dry conditions. However, the GR24 application reduced this increase to a certain extent. The most notable reduction in MDA content was demonstrated with 0.1 mg L^−1^ GR24, compared to other levels of GR24 under drought as well as well-watered conditions (Figure 3). When plants were exposed to drought stress. The H_2_O_2_ contents were radically enhanced to 92.4% in HY-1898 and 74.5% in FH-1046 compared to well-watered maize seedlings. Increased production of H_2_O_2_ in dry-stressed blooms was significantly inhibited by GR24 foliar application in a dose-dependent manner (Figure 3). Treatment with 0.1 mg L^−1^ GB decreased drought-induced stress by limiting H_2_O_2_ up to 37.6% in HY-1898, and 21.7% in FH-1046 cultivars. However, other concentrations of GR24 also significantly mitigated the negative effects of drought in both maize cultivars.

Plants use many strategies to escape oxidative damage, including activating antioxidant defense mechanisms. Various antioxidant enzymes, including SOD, POD, and CAT, play essential roles in these efforts. As shown in Figure 3, drought stress increased SOD, CAT, and POD activities compared to control plants by 37.8, 93.5, and 92.3%, respectively. This increase was further improved with foliar application of GR24 in a dose-dependent manner (Figure 3). The SOD activity progressively increased with increasing concentration of GR24 in HY-1898 (62.8, 46.7, and 79.1%) and FH-1046 (56.7, 66.3, and 91.4%) under drought stress. Similarly, 0.01 mg L^−1^ foliar application significantly mitigated drought-induced stress by enhancing CAT (270.8%) and POD (210.4%) activities in both maize seedlings (Figure 3).

### 3.3. The effects of exogenous GR24 on osmolytes of two maize hybrids

**Figure 4.**
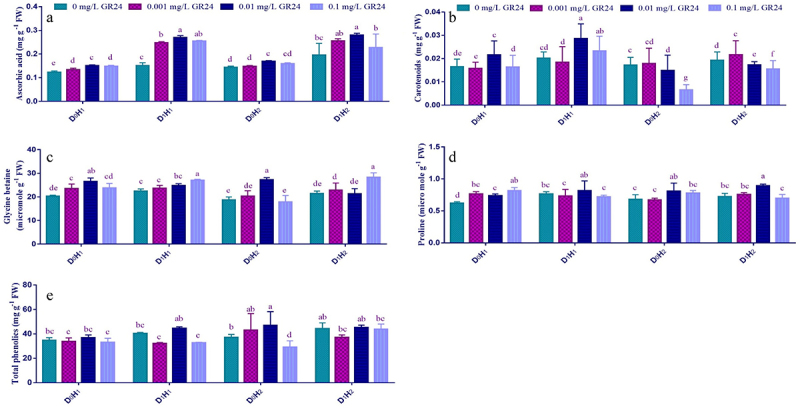
Effect of SL (GR24) application on ascorbic acid (a), carotenoids (b), glycine-betaine (c), proline (d), and total phenolics (e) of two maize hybrids under drought stress. Different lowercase letters indicate significant differences as determined by the LSD test (*p*≤ 0.05). Replication = 3; D_0_= no drought control; D_1_= drought stress at 60% field capacity; H_1_= maize hybrid (FH-1046); H_2_= maize hybrid (HY-1898).

GR24 functions as a potent signaling molecule and protects plants from abrasive oxidative stress. Significant changes in the activities of ascorbic acid, carotenoids, glycine betaine (GB), proline and total phenolics were observed in the drought treated maize hybrid plants with and without GR24 foliar application (Table S1; Figure 4). The exogenous application of GR24 in the form of foliar spray, though considerably increased all the osmolytes of current investigation in 100% irrigation regime; however, the increase of these osmolytes were many folds with 60% irrigation. Among various concentrations tested against drought stress, 0.01 mg L^−1^ foliar application causes an increase of 107.13, 68.17, 22.50, 5.89 and 10.13% in while an increase percentage was 133.25, 1.61, 5.52, 24.11 and 54.59% in H_2_D_1_ maize hybrids, respectively, for AsA (Figure 4a), carotenoids (Figure 4b), GB (Figure 4c), proline (Figure 3d) and total phenolics (Figure 3e). Both maize hybrids show non-significant (*P* > 0.05) difference of studied osmolytes either under drought stress or with GR24 foliar application except carotenoids (Table S1).

### 3.4. The effects of exogenous GR24 on yield of maize hybrids

**Figure 5.**
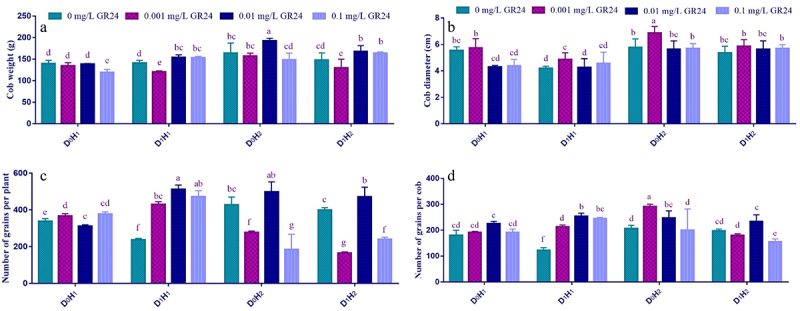
Effect of SL (GR24) application on cob weight (a), cob diameter (b), number of grains per plant (c), and number of grains per cob (d) of two maize hybrids under drought stress. Different lowercase letters indicate significant differences as determined by the LSD test (*p*≤ 0.05). Replication = 3; D_0_= no drought control; D_1_= drought stress at 60% field capacity; H_1_= maize hybrid (FH-1046); H_2_= maize hybrid (HY-1898).

Data of grain yield of maize hybrids under drought stress conditions revealed that strigolactone (GR24) application has a significant effect on these parameters (Table S1). The drought stress (D_1_) triggered a remarkable reduction in cob diameter (3.39%), number of grains per plant (17.50%) and number of grains per cob (20.01%), compared to non-stressed (D_0_) in both maize hybrids (Figure 5). Under the drought-stressed condition, the 0.01, and 0.1 mg L^−1^ GR24 spray increased the cob weight by 9.32, 8.59% (Figure 5a), cob diameter by 15.87, 8.67% (Figure 5b), number of grains per plant by 115.71, 98.18% (Figure 5c), and number of grains per cob by 107.90, 100.55% (Figure 5d) in H_1_. Maximum yield, and its related components were observed from the plants which were treated with 0.01 mg L^−1^ followed by 0.1 mgL^−1^ and 0.001 mg L^−1^ GR24 as foliar spray (Figure 5). More importantly, both maize hybrids show a significant difference for all yield-related attributes studied in current investigation.

### 3.5. Multivariate analysis

**Figure 6.**
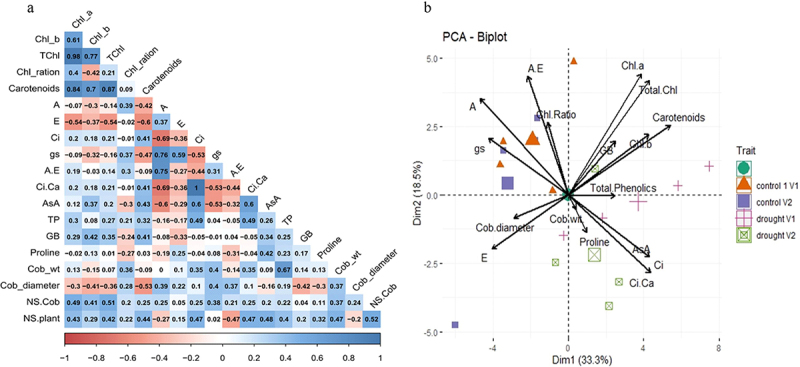
Pearson correlation (a) and principal components analysis (PCA) (b) biplot between various attributes of maize hybrids revealed the impact of foliar application of synthetic analogue of strigolactones (GR24) under drought stress on physiological and biochemical aspects. Chl_*a*: chlorophyll *a*; Chl_*b*: chlorophyll *b*; TChl: total chlorophyll; Chl_ratio: chlorophyll ratio; carotenoids; *A*: net photosynthetic rate; *E*; transpiration rate; *C*_i_: internal CO_2_ concentration; *g*_s_: stomatal conductance; A.E: water use efficiency; *C*_i_.*C*_a_: ratio of leaf intrinsic CO_2_ concentration to ambient CO_2_ concentration; AsA: leaf ascorbic acid; TP: total phenolics; GB: Glycinebetaine; proline; Cob_wt: cob weight; Cob_diameter; NS.Cob: number of seeds per cob; NS.Plant: number of seeds per plant (***≥0.450; **≥0.360; *≥0.325; ns ≤ 0.325).

For the investigation of physiological, biochemical and yield parameters with SLs and drought in maize hybrids, we performed Pearson correlation and principal component analysis (PCA). Positive and negative Pearson correlation was recorded among various attributes in two maize hybrids under drought conditions with three doses of GR24. It was assessed that, there was significant correlation between photosynthetic pigments, cob weight with total phenolics, and glycine betaine with photosynthetic pigments. *Chl* contents have negative correlation with many gas exchange attributes (*A, E, g*_*s*_), proline and cob diameter (Figure 6). Carotenoid contents showed a positive correlation to all the attributes except gas exchange attributes, cob weight and cob diameter. Internal CO_2_ concentration (*Ci*) showed negative correlation with *A* and *E*. Ascorbic acid (AsA) concentration is highly positively correlated with *Ci/Ca*. Among yield attributes, number of seeds per plant/seed yield per plant showed a highly positive correlation to all other attributes except *A, A/E* and cob diameter (Figure 6).

This correlational expression pattern among different plant attributes of two *Z. mays* hybrids is further verified by PCA-Biplot. Figure 6 exhibited PCA-Biplot of different features of *Z. mays* hybrids under drought stress with strigolactone foliar application. Principal component for physico-chemical traits showed a cumulative contribution rate of 33.3 ~ 8.5%. Chlorophyll pigments showed eigenvalues higher than 2.5 and represent a comprehensive close relationship. Both Dim 1 and Dim2 jointly presented 33.3% and 18.1% variability in data. Dim1 clearly demonstrates a distinction of maize attributes after exposure to drought stress compared to control relatives. In the dataset, it is further confirmed, as was previously showed in correlation matrix that proline, *Ci/Ca*, AsA, total phenolics *Chl b*, *total Chl*, GB, carotenoids, cob weight were closely placed and showed a significant positive relationship among themselves but negatively correlated with gas exchange attributes (Figure 6). It clearly demonstrates that variables in control plants did not significantly match with the same variables in drought treated maize hybrid plants. Furthermore, Table 1 presents a comparative analysis of the respective parameter responses in two distinct hybrids.

**Table 1.** Comparative account of the parameters as response in two different hybrids.

Attributes	Response	
	FH-1046	HY-1898
Chl a	Better response to 0.01 mg/L SL (D_1_H_1_)	
Chl b	Better response to 0.01 mg/L SL (D_1_H_1_)	
Chl a/b ratio	Non-significant difference	Non-significant difference
Total Chl	Better response to 0.01 mg/L SL (D_1_H_1_)	
*A*	Non-significant difference with 0.001 and 0.01 mg/L SL (D_0_H_1_, D_1_H_2_)	Non-significant difference with 0.001 and 0.01 mg/L SL (D_0_H_1_, D_1_H_2_)
*Ci*	0.01, 0.1 mg/L SL concentration showed improvement (D_1_H_1_)	0.01, 0.1 mg/L SL concentration showed improvement (D_1_H_1,_ D_1_H_2_)
*Ci/Ca*	0.01, 0.1 mg/L SL concentration showed improvement (D_1_H_1_)	0.01, 0.1 mg/L SL concentration showed improvement (D_1_H_1,_ D_1_H_2_)
*E*		Transpiration rate decreased with 0.01 mg/L SL concentration (D_0_H_2,_ D_1_H_2_)
*G*_*s*_	Increased with 0.001, 0.1 mg/L concentration of SL (D_0_H_1,_ D_0_H_2_)	Increased with 0.001, 0.1 mg/L concentration of SL (D_0_H_1,_ D_0_H_2_, D_1_H_2_)
EL		Decreased with 0.01 mg/L concentration of SL (D_1_H_2_)
MDA	Decreased with 0.01 mg/L concentration of SL (D_0_H_1,_ D1H_1_)	Decreased with 0.01 mg/L concentration of SL (D_0_H_2_, D_1_H_2_)
H_2_O_2_	Increased with 0.1 mg/L concentration of SL (D_0_H_1,_ D1H_1_)	Increased with 0.1 mg/L concentration of SL (D_0_H_2_, D_1_H_2_)
CAT	Increased with 0.1 mg/L concentration of SL (D_0_H_1,_ D1H_1_)	Increased with 0.1 mg/L concentration of SL (D_0_H_2_, D_1_H_2_)
POD	Increased with 0.1 mg/L concentration of SL (D_0_H_1,_ D1H_1_)	Increased with 0.1 mg/L concentration of SL (D_0_H_2_, D_1_H_2_)
SOD	Increased with 0.1 mg/L concentration of SL (D_0_H_1,_ D1H_1_)	Increased with 0.1 mg/L concentration of SL (D_0_H_2_, D_1_H_2_)
AsA	Increased with 0.01 mg/L concentration of SL (D_1_H_1_)	Increased with 0.01 mg/L concentration of SL (D_1_H_2_)
Carotenoids	Increased with 0.01 mg/L concentration of SL (D1H_1_)	Increased with 0.001 mg/L concentration of SL (D_1_H_2_)
GB	Increased with 0.1 mg/L concentration of SL (D1H_1_)	Increased with 0.1 mg/L concentration of SL (D_1_H_2_)
Proline	Increased with 0.1 mg/L concentration of SL (D1H_1_)	Increased with 0.1 mg/L concentration of SL (D_1_H_2_)
Total phenolics	Increased with 0.1 mg/L concentration of SL (D_1_H_1_)	Increased with 0.1 mg/L concentration of SL (D_1_H_2_)
Cob weight		Increased with 0.01 mg/L concentration of SL (D_0_H_2_, D_1_H_2_)
Cob diameter	Increased with 0.1 mg/L concentration of SL (D_0_H_1,_ D_1_H_1_)	Increased with 0.1 mg/L concentration of SL (D_0_H_2_, D_1_H_2_)
Number of grains per plant	Increased with 0.1 mg/L concentration of SL (D_0_H_1,_ D_1_H_1_)	Increased with 0.1 mg/L concentration of SL (D_0_H_2_, D_1_H_2_)
Number of grains per cob	Increased with 0.1 mg/L concentration of SL (D_0_H_1,_ D_1_H_1_)	Increased with 0.1 mg/L concentration of SL (D_0_H_2_, D_1_H_2_)

## 4. Discussion

The current study supports our hypothesis that foliar application of SLs (GR24) helps to mitigate drought stress in maize hybrids by improving chlorophyll contents, gas exchange activity, and osmolyte production in plants grown under drought stress. Since the discovery of SLs’ hormonal activity in 2008, there has been significant development in the field of SLs research. Several significant advances have been made in our understanding of SLs biosynthesis, transport, and perception. The revelation of a new class of plant hormones and the long-desired SL biosynthetic and response mutants discovered SL’s hormonal function extremely valuable.^38^ This phytohormone regulates and activates developmental processes via interacting signaling networks, which help the plants to acclimatize against biotic and abiotic stresses and, consequently, their survival.^39^ In *Arabidopsis thaliana* and rice plants, many proteins including DWARF14 (D14), MORE AXILLARY GROWTH 2 (MAX2), SUPPRESSOR OF MAX2-LIKE 6, 7 and 8 (SMXL6, SMXL7 and SMXL8) their orthologues make complex upon SLs signal sensing and display a critical role in SLs signaling.^40–42^ An increasing amount of evidence shows that cytokinin (CKs) and SLs play key roles in plant drought acclimation, making CKs and SLs a ‘primary topic’ in this academic field.^43^ The cross-talk of SLs and CK at the signaling level, particularly related to stomatal closure during drought circumstances, is poorly understood. Increased SL concentration in shoots has been anticipated to increase plant sensitivity to ABA, lowering stomatal conductance and increasing plant survival.^44^ SLs also coordinate changes in the homeostasis of resource acquisition through strategic plant development, allowing plants to respond to nutrient deprivation. Instead of acting alone, SL interacts with abscisic acid, cytokinin, auxin, ethylene, and other plant phytohormones to generate complex signaling networks.^45^ In our understanding, the important components and their roles in significant achievements have been seen for jasmonates, salicylic acid, abscisic acid, auxin, gibberellic acid, brassinosteroids, and ethylene plant responses against the various abiotic stress.^31^ Drought is a multidimensional constraint that modifies plants’ physiology and morphology and causes numerous devastating changes in biochemical pathways. Plant biomass comprises about 80–95% water, vital in maintaining structural and functional integrity.^46^

Drought stress causes impairment in the plants’ molecular, physicochemical, and morphological processes.^47^ Chlorophyll pigments are the key components of the photosynthetic machinery of plants that impart a significant role in light harvesting and generation of reducing agents, and their production level shows their photosynthetic abilities.^48^ SL might be involved in regulating genes involved in light harvesting^49^ in tomatoes (*Solanum lycopersicum*) and changing the physiology of photosynthetic pigments for the energy-capturing ability of the leaf of Arabidopsis.^49^ Under stress conditions, SL can also increase plant stomatal conductance (Gs), transpiration rate (Tr), and intercellular CO_2_ concentration (Ci), hence boosting the net photosynthetic rate (Pn).^50^ SL can also help with photosynthesis by maintaining the stability of the photosystem II (PSII) super complex, increasing D1 protein turnover, photosynthetic electron transport, and the demand for ATP and NADPH in the dark cycle, and increasing photosystem efficiency, all of which promote plant growth and development.^20,21,51^ Research on Max1 and Max2 mutants of Arabidopsis demonstrated that SL may either separately or with ABA produced drought tolerance.^52^ Mitigation of chlorophyll content might have occurred due to the loss and disorganization of the chloroplast membrane and the formation of lipid droplets.^53^ Limiting ambient CO_2_ entrance to the carbon dioxide fixation site could also induce stomatal closing, decreasing the photosynthetic mechanism.^54^ SLs mitigate drought stress by improving the gas exchange abilities in grape wine (*Vitis vinifera* L.).^55^ This might be due to the up-regulation of F- box Max2 proteins, which are involved in the biosynthetic pathway of SLs and ABA signaling; hence, ABA signaling is an important indicator of drought stress that regulates the stomatal regulation, which controls the gas exchange through stomata.^45^ Similar investigations were examined in maize seedlings priming with growth promoters.^56^

Studies have declared that SL mutant plants showed less information about the signaling and gene regulation of strigolactones, which correlated with stress tolerance mechanism in many plant species, while exogenous treatment of a synthetic strigolactone analogue triggered drought stress tolerance in *Arabidopsis* and wheat (*Triticum aestivum* L.).^57^ Leaf ascorbic acid and total phenolics are important non-enzymatic antioxidants that detoxify the free radicals and boost plant immunity to various stresses.^58^ Phenolics and ascorbic acid are produced in plants as secondary metabolites, which play a scavenging role in plants to avoid the detrimental effects of oxidative stress. In the current study, the exogenous application of GR24 considerably improved the leaf ascorbic acid and total phenolic contents under drought conditions. These were like Min et al.,^55^ in grapevine, demonstrating that ascorbic acid value increased by GR24 in maize seedlings. In the current experiment, most of the features we analyzed depicted that the SL application had a greater positive influence on plants under drought conditions.^57^ The exact mechanism that how GR24 regulates the leaf ascorbic acid and total phenolics is still unknown. Phenolics are correlated with stress-tolerant antioxidants because these compounds are redox-sensitive.^59^ They break the chain reaction due to their high donor ability of electron pair and make these phenolic contents perfect for antioxidant activity.^60^

Organic osmolytes (glycine-betaine and proline) are important osmoprotectants that reduce the harmful effects of drought. Hyperaccumulation of osmoprotectants in the cell is an important indicator of stress tolerance in plants,^61^ which is the capability to overwhelm water and osmotic stress.^62^ Under drought stress, accumulation of osmoprotectants helps the plants to tolerate the stress level.^63^ Our investigation revealed that GR24 increased the concentration of glycine-betaine and free proline. These results coincided with findings in apples by Zheng et al.^64^ Previous studies on maize exhibited that applying growth promoters^65^ as an external source further improves the osmotic adjustment capacity by regulating the osmo-regulators under water-deficient stress.^66^ Nutrient acquisition, particularly under phosphorus deficit, influences SLs excretion and transportation in agricultural soils.^67^ The improvement in SLs content corresponds to the expression of SLs biosynthesis genes in rice roots, which is higher under nitrogen (N) and/or P-limiting conditions than under-regulated normal growth settings.^68^ Consequently, under drought stress increased level of GB has a protective role against oxidative stress by stabilizing membranes and enzymes.^69^ Proline accumulation in maize works as an osmo-protectant and stabilizes the plants’ photosynthetic machinery from ROS.^69^ Glycine-betaine produce under drought stress predominantly in young tissues, protects and stabilizes the cellular membranes and organelles from free radicals.^70^

**Figure 7.**
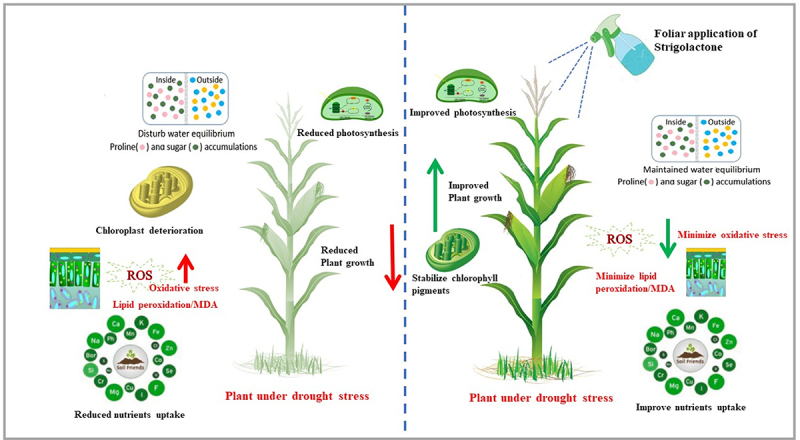
A theoretical scheme represents two different mechanisms of maize plants under drought stress (60% field capacity) with and without foliar application of synthetic analogue of strigolactones (GR24). Drought stress adversely affects the morphological, biological, and physiological characteristics of the maize plant, resulting in minimizing the uptake of the essential nutrients, disturbing water equilibrium, enhancing the oxidative stress, and deteriorating the organelles’ structure, i.e., mitochondria and chloroplast, which ultimately reduced the photosynthesis activity and plants growth. The plant cells are not adequately protected by this stress-responsive mechanism, which causes excessive MDA release from reactive oxygen species-damaged cells. The situation can be the exact opposite in the case of maize plants whose leaves have been exposed to the foliar application of synthetic analogue of strigolactones (GR24). GR24 induced stomatal closure to prevent evapotranspiration and mediate stress-responsive pathways to lessen cell damage during drought stress, which helps to enhance and stabilize the chlorophyll pigments and osmolytes accumulation, which results in improved photosynthesis activity, reduced oxidative stress, and ultimately improved the yield of the plant.

Figure 7 elucidates the contrasting mechanisms in maize plants subjected to 60% field capacity drought stress, with and without the foliar application of a synthetic analogue of SL. Under drought stress, the SL counterpart AB01 increased maize grain yield and kernel weight.^71^ Mutants lacking SL biosynthesis or signaling are hypersensitive to adverse environmental circumstances such as drought, salt, and osmotic stress.^64,72,73^ Current experimental data demonstrated that yield-related attributes showed negative results on exposure to drought stress. According to de Souza et al.^74^ in maize which showed yield loss by reducing the grain weight and the number of grains per ear under drought stress. Yield loss under severe drought might be due to the decreased leaf area and impairment of the chlorophyll pigments.^75^ GR24 application considerably increased the cob diameter. Furthermore, the number of seeds per cob and plant increased under drought stress. The evidence suggests that when water was removed from the experimental pots, SLs application had a stronger favorable influence in both maize hybrids. Furthermore, Zwanenburg and Mwakaboko^76^ established that SL mimics SA in the laboratory experiment. Since it is a non-monitoring method with enormous practical application in dynamic field situations.^77^ Still, we must rely on more than one trial on one crop to establish any recommendation at the field level. More extensive experimentation might be helpful, such as hormone biosynthesis, cross-talk, and gene expression on various crops. An absolute investigation of this phytohormone’s molecular interactions could be explored.

## 5. Conclusion

This study suggests that applying the synthetic analogue GR24, a type of SLs, can effectively mitigate drought stress in maize hybrids. GR24 application improves photosynthesis, osmotic adjustment, and antioxidant defenses in maize plants facing water scarcity. It also enhances yield-related parameters, like cob diameter and seed production, offering promise for crop productivity in drought-prone areas. For future directions, research should focus on optimizing the application of GR24 in practical farming settings. This includes investigating dosages, timing, and application methods. Expanding the study to diverse maize hybrids and other crops will provide a broader perspective. Understanding SL-mediated drought tolerance’s molecular mechanisms can lead to more targeted crop improvement strategies. Large-scale field trials across different regions are essential to validate these findings in real-world conditions. Additionally, assessing the environmental impact of the GR24 application is crucial for sustainable agriculture. Combining SL application with other drought mitigation techniques and developing cost-effective formulations will enhance its utility in farming. In summary, GR24 holds promise as a tool to enhance drought resilience in agriculture. Further research and practical implementation can contribute to sustainable farming practices, bolstering crop yield and food security in the face of water scarcity.

## Supplementary Material

Supplemental Material
