# Supplementary material for: Effect of strigolactone on growth, photosynthetic efficiency, antioxidant activity, and osmolytes accumulation in different maize (Zea mays L.) hybrids grown under drought stress
Source: Plant Signal Behav. 2023 Dec 17;18(1):2262795. doi: 10.1080/15592324.2023.2262795 (PMC10730227; doi:10.1080/15592324.2023.2262795)
Supplement: Supplemental Material [file KPSB_A_2262795_SM1340.docx]

**Table S1:** Analysis of variance for the influence of foliar application of synthetic analogue of strigolactones (GR24) on different plant traits under drought stress

| **Source of Variation** | **Df** | **Chl *a*** | **Chl *b*** | **Total Chl.** | **Chl *a/b* ratio** | **Carotenoids** | ***A*** | ***E*** |
| --- | --- | --- | --- | --- | --- | --- | --- | --- |
| Hybrid (H) | 1 | 0.149** | 0.028*** | 0.291** | 0.159ns | 0.0002** | 18.656* | 0.372* |
| Drought (D) | 1 | 0.003ns | 0.009* | 0.019ns | 0.618ns | 0.0002** | 345.007*** | 1.370*** |
| GR24 | 3 | 0.108** | 0.008** | 0.169*** | 3.040* | 0.0001ns | 21.931** | 0.0175ns |
| H x D | 1 | 0.077* | 0.006ns | 0.0353ns | 10.822*** | 0.0001ns | 0.530ns | 0.003ns |
| H x GR24 | 3 | 0.154*** | 0.009** | 0.238*** | 0.816ns | 0.0001* | 5.023ns | 1.572*** |
| D x GR24 | 3 | 0.045ns | 0.007404ns | 0.056ns | 0.551ns | 0.0002ns | 8.352ns | 0.289* |
| H x D x GR24 | 3 | 0.006ns | 0.008** | 0.014ns | 5.159ns | 0.00001ns | 21.709** | 0.029ns |
| Error | 32 | 0.017 | 0.002 | 0.023 | 0.749 | 0.0002 | 3.904 | 0.073 |
| **Source of Variation** | **df** | ***g_s_*** | ***C_i_*** | ***A/E*** | ***C_i_/C_a_*** | **Leaf AsA** | **Total Phenolics** | **Glycine betaine** |
| Hybrid (H) | 1 | 533.333* | 11451.541** | 9.273ns | 0.092** | 0.002* | 277.922** | 36.815** |
| Drought (D) | 1 | 8008.3333*** | 76976.101*** | 76.682*** | 0.621*** | 0.093*** | 121.285* | 35.177** |
| GR 24 | 3 | 427.778* | 1955.624ns | 29.593*** | 0.016ns | 0.009*** | 168.589** | 35.177*** |
| H x D | 1 | 75ns | 11731.253** | 3.251ns | 0.095** | 0.001.333ns | 1.687ns | 6.289ns |
| H x GR24 | 3 | 472.222ns | 1589.678ns | 38.249*** | 0.013ns | 0.008.056ns | 9.589ns | 0.766ns |
| D x GR24 | 3 | 247.222ns | 8264.380*** | 11.352** | 0.067*** | 0.004*** | 74.873ns | 57.425*** |
| H x D x GR24 | 3 | 247.222ns | 8184.096*** | 6.953ns | 0.066*** | 0.004.5ns | 80.957* | 16.616** |
| Error | 32 | 125 | 1152.237 | 2.440 | 0.009 | 0.0039225 | 26.738 | 2.976 |
| **Source of Variation** | **df** | **Proline** | **Cob weight** | **Cob diameter** | **Number of seeds per cob** | **Number of seeds per plant** |  |  |
| Hybrid (H) | 1 | 0.0024224ns | 6192.791*** | 14.192*** | 1397.521ns | 500.521ns |  |  |
| Drought (D) | 1 | 0.009ns | 40.940ns | 2.210** | 3485.021* | 62424.188*** |  |  |
| GR 24 | 3 | 0.029** | 1281.452*** | 1.809** | 8967.854*** | 53432.021*** |  |  |
| H x D | 1 | 0.0031831ns | 1560.774** | 0.075ns | 9436.021*** | 121907.52*** |  |  |
| H x GR24 | 3 | 0.008ns | 359.682ns | 0.241ns | 4857.354*** | 86540.743*** |  |  |
| D x GR24 | 3 | 0.021** | 1092.326*** | 0.896* | 2121.743* | 13272.187*** |  |  |
| H x D x GR24 | 3 | 0.006ns | 136.910ns | 0.207ns | 4827.188*** | 18813.632*** |  |  |
| Error | 32 | 0.005 | 130.249 | 0.280 | 593.062 | 1325.5 |  |  |

*, ** and *** = significant at 0.05, 0.01 and 0.001 levels, ns = non-significant respectively, ***Chl a***= chlorophyll *a*; ***Chl* *b***= chlorophyll *b*; **Total *Chl***=total chlorophyll; ***Chl* *a/b* ratio**=chlorophyll *a/b* ratio; ***A*** = net photosynthetic rate; ***E*** = transpiration rate; ***Ci***=internal CO_2_ concentration; ***g_s_*** = stomatal conductance; ***A*/*E*** = Water use efficiency; ***C_i_*/*C_a_*** = ratio of leaf intrinsic CO2 concentration to ambient CO_2_ concentration, **Leaf AsA**=leaf ascorbic acid.
